# Discovery of AMG 133, a Glucose-Dependent Insulinotropic Polypeptide Receptor Antagonist and Glucagon-Like Peptide 1 Receptor Agonist Antibody-Drug Conjugate for the Treatment of Obesity

**DOI:** 10.1021/acs.jmedchem.6c00032

**Published:** 2026-04-06

**Authors:** Bin Wu, James R. Falsey, Chawita Netirojjanakul, Brad Herberich, Jerry Ryan Holder, Kelvin Sham, Jennifer Aral, Neil Forsythe, Kenneth W. Walker, Shu-Chen Lu, Todd Hager, Shanaka Stanislaus, Renee Komorowski, Larissa Atangan, Michal Achmatowicz, Dante Romanini, Dohan Weeraratne, Les P. Miranda, Murielle M. Véniant, Yuan Cheng

**Affiliations:** 1 Therapeutic Discovery, Amgen Research, One Amgen Center Dr, Thousand Oaks, California 91320, United States; 2 Department of Cardiometabolic Disorders, Amgen Research, One Amgen Center Dr, Thousand Oaks, California 91320, United States; 3 Translational Safety & Bioanalytical Sciences, Amgen Research, One Amgen Center Dr, Thousand Oaks, California 91320, United States; 4 Process Development, Amgen Inc. One Amgen Center Dr, Thousand Oaks, California 91320, United States

## Abstract

Multispecific therapeutics represent an increasingly important approach for enhancing the efficacy in complex diseases. Here, we report the design and optimization of novel antibody-peptide conjugates that combine glucose-dependent insulinotropic polypeptide receptor (GIPR) antagonism with glucagon-like peptide 1 (GLP-1) receptor (GLP-1R) agonism for the treatment of obesity. A series of hybrid molecules was generated by conjugating synthetic GLP-1 peptides to IgG-based anti-GIPR antibodies, yielding markedly prolonged systemic exposure of the structurally intact GLP-1 peptide. In diet-induced obese mice and obese monkeys, once weekly administration of anti-GIPR-Ab/GLP-1 conjugates produced sustained body weight loss and improvements in metabolic parameters. This optimization effort culminated in the discovery of AMG 133, currently in phase III clinical trials with a profile that may support monthly dosing.

## Graphical abstract

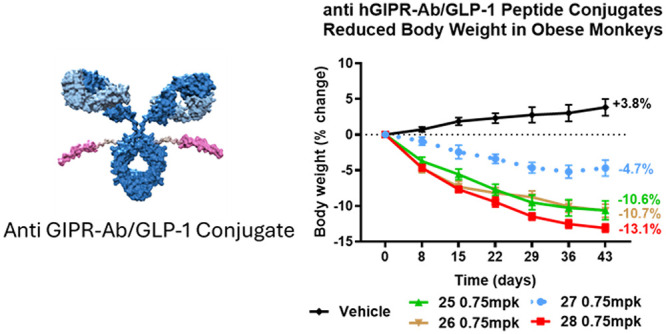

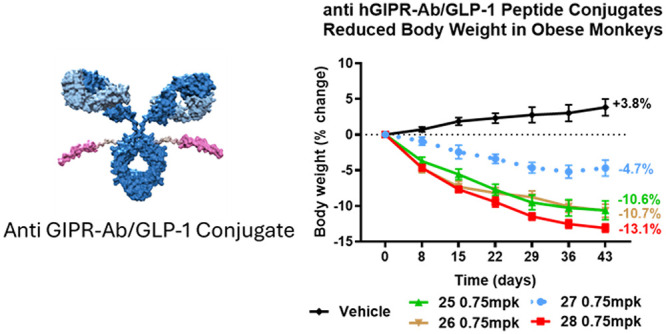

## Introduction

Obesity is one of the most important public health issues, with nearly 40% of US adults and over two billion people worldwide being affected. Obesity and its associated comorbidities, including heart disease, stroke, type 2 diabetes, and certain types of cancer, are among the leading causes of premature death.^1,2^ Lifestyle modification remains the primary approach for obesity management, but for most patients, it yields modest and often nondurable weight loss. As a result, surgical interventions such as bariatric surgery, along with the treatment of GLP-1R agonists,^3,4^ have become the principal therapeutic options with potential to improve obesity-related conditions. While surgery can be highly effective, a proportion of patients experience weight regain over several years.^5^ GLP-1R agonists have shown substantial promise, inducing clinically meaningful body weight loss by reducing food intake and delaying gastric emptying.^6^ Currently, two GLP-1R agonists, Liraglutide and Semaglutide, have been approved for weight management in obesity. Liraglutide and Semaglutide are both lipid-conjugated peptide drugs administered daily and weekly, respectively, and have demonstrated clinically relevant weight loss of ≥ 5%. However, side effects such as nausea, vomiting, and gastrointestinal issues have been reported as the most common adverse effects.^7,8^ Combining the activity of other regulatory hormones with GLP-1R agonism into a single peptide molecule has emerged as another promising approach for effective weight loss.^9–13^ Several coagonists of GLP-1R/glucagon receptor (GCGR), GLP-1R/GIPR, as well as triagonists of GLP-1R/GCGR/GIPR have been rationally designed and tested in preclinical models of obesity before advancing to clinical trials. In particular, Tirzepatide, a lipid-conjugated dual GIP/GLP-1 receptor agonist, has demonstrated substantial clinical efficacy for weight reduction^13^ and has been approved for chronic weight management. Like other lipid conjugates, Tirzepatide is administered once weekly.

Through multiple genetic studies performed in humans, genetic variants found in Japanese, European, and North American populations show that lower expression or function of GIPR is associated with a lower body mass index (BMI).^14,15^ Additionally, multiple knockout mouse models, including whole-body GIPR knockout, central nervous system-specific GIPR knockout, and GIP knockout in intestinal K cells, have shown protection against diet-induced obesity (DIO).^16–18^ We previously reported the development of anti-GIPR antibodies as a therapeutic approach for the treatment of obesity. In DIO mice, treatment with an antimouse GIPR antibody (anti-mGIPR-Ab) prevented body weight gain, whereas in obese NHPs, an antihuman GIPR antibody (anti-hGIPR-Ab) monotherapy reduced body weight. We observed an enhanced effect on weight loss in DIO mice when the anti-mGIPR-Ab was combined with Liraglutide, a human GLP-1 analog that is also active on mouse GLP-1R, owing to human-mouse GLP-1 sequence conservation, resulting in 23.5% weight reduction.^19^ Similarly, in obese NHPs, combining the anti-hGIPR-Ab with Dulaglutide, a once weekly injectable GLP-1 Fc fusion protein, led to 14.5% weight loss.^20^ Results from the combination studies supported the development of a bispecific GIPR-Ab and GLP-1R agonist peptide conjugate, which would combine GIPR antagonism and GLP-1R agonism in a single molecule. In proof-of-concept (POC) studies, administration of such bispecific molecules produced significant weight loss and improvements in metabolic parameters in obese mice and NHPs.^21^

Here, we describe the discovery, optimization, and preclinical development of antibody-peptide conjugates that couple an anti-GIPR antibody with a GLP-1R agonist peptide. These hybrid constructs employ a modular architecture, in which a chemically synthesized GLP-1 analogue is site-specifically tethered to a GIPR antibody through a defined linker, enabling independent tuning of the antibody, linker, and peptide components. A key objective of the optimization campaign was to extend systemic exposure of the GLP-1 component and to align its half-life and pharmacodynamic duration with those of the parental anti-GIPR antibody, thereby enabling a repeat dosing regimen that maintains a consistent coverage of both GLP-1R and GIPR.

The native GLP-1 peptide (1) has a short plasma half-life (1.5–5 min), which limited earlier GLP-1 therapies in the clinic.^22^ To overcome rapid clearance and DPP-4-mediated degradation, FDA-approved GLP-1 therapeutics employ several half-life extension strategies, including DPP-4-resistant sequence modifications, lipidation (e.g., Liraglutide and Semaglutide), direct albumin fusion (Albiglutide), and Fc fusion (e.g., Dulaglutide).^23–25^ Among long-acting modalities, antibody-peptide conjugation (or genetic fusion to an antibody) can provide substantial half-life extension by increasing the hydrodynamic size to reduce renal filtration and enabling neonatal Fc receptor (FcRn)-mediated antibody recycling. In this context, we developed GIPR-Ab/GLP-1 conjugate using a previously reported site-specific conjugation approach,^26,27^ yielding homogeneous products bearing two copies of the GLP-1 analog per antibody via engineered cysteine handles. The GLP-1 moiety was chemically synthesized to enable incorporation of noncanonical residues for improved metabolic stability and rational design modifications to tune potency. Using this platform, we prepared a series of mGIPR-Ab and GLP-1 conjugates and evaluated them *in vitro* and *in vivo* to investigate how conjugation site, linker composition, and peptide modifications influence pharmacokinetic profile and weight loss efficacy.

Through iterative refinement, we have identified unimolecular, dual-targeting GIPR-Ab/GLP-1 conjugates that exhibited balanced activity at each constitutive receptor and attractive pharmacokinetic properties. Mouse receptor cross-reactive conjugates demonstrated robust *in vivo* efficacy in reversing diet-induced obesity (DIO) in rodents. In the obese NHP model, four conjugates with hGIPR-Ab were studied, and conjugate **28** (AMG 133) demonstrated 16.9% weight loss compared to the vehicle group after 6 weeks of weekly subcutaneous dose at 0.75 mg/kg. These GIPR-Ab/GLP-1 bispecific compounds also reduced fasting insulin and normalized lipid metabolism.

## Results

### GIPR-Ab/GLP-1 Conjugate Design and Synthesis

As previously reported,^19^ we had identified a mGIPR-Ab with hu IgG1 SEFL2 backbone (**2**, mGIPR IC_50_: 6.2 nM) that was active in mice and a hGIPR-Ab (**3**) with optimized overall properties on the human receptor.^28^ We used mGIPR-Ab to conjugate with GLP-1 peptide analogs for the *in vivo* studies in mice, and hGIPR-Ab conjugates for the *in vivo* studies in NHPs. Conjugation was achieved through a C-terminal bromoacetamide on the linker,^29^ which chemoselectively alkylated the engineered cysteine thiols to form a stable thioether linkage.^30^ The site of conjugation is important as it can significantly impact the stability, activity, and pharmacokinetic profiles of the conjugation product.^31,32^ We had screened a large panel of surface-exposed cysteine mutation sites and identified multiple positions that were compatible with the conjugation method.^26^ Three conjugation sites were selected for mGIPR-Ab to investigate their effects on the properties of GIPR-Ab/GLP-1 conjugates. These three sites are located at different regions of the antibody, such as D88C on the light chain near the N-terminal region, T487C on the Fc near the C-terminal region, and E384C on the Fc close to the hinge region (Figure 1A). The D88C, E384C, and T487C cys-mAb of mGIPR-Ab were prepared, respectively, to conjugate with GLP-1 peptide analogs.

**Figure 1:**
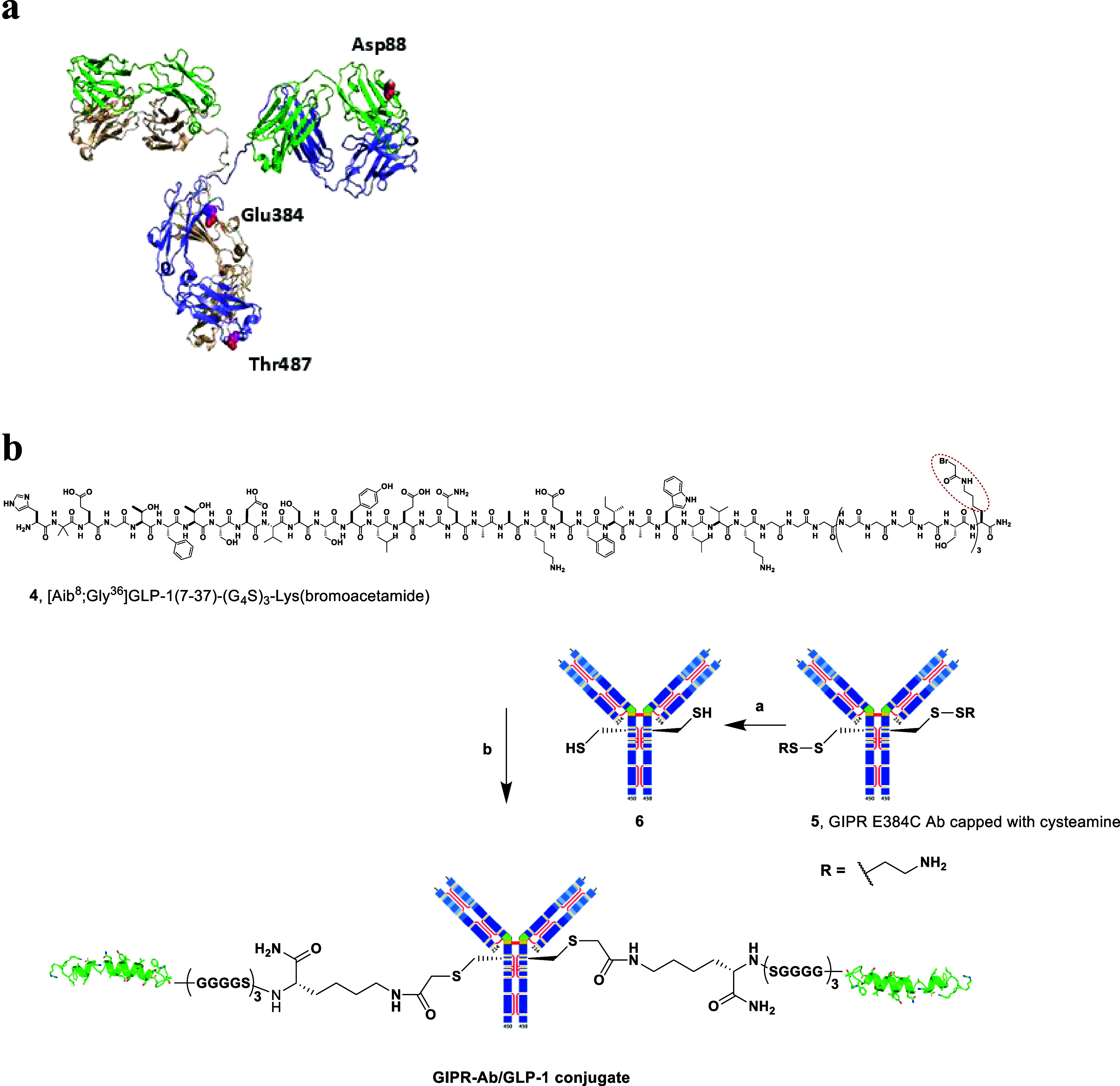
(a) Representation of GIPR Antibody scaffold with 3 cysteine mutation sites (highlighted in fuchsia) were evaluated. (b) Conjugation synthesis exemplified with (G_4_S)_3_K linked peptide and GIPR E384C Ab: (a) Antibody **6** with free thiol groups was generated from **5** with cysteamine-capped engineered cysteine via redox conditions. (b) Conjugation of peptide **4** (bromoacetamide highlighted in the red circle) at the engineered cysteines of **6**.

A representative conjugation protocol is shown in Figure 1B, in which a 2-aminoisobutyric acid (Aib) modified GLP-1 analogue **4** [Aib^8^;G^36^]­GLP-1­(7–37) with a (G_4_S)_3_K linker at the C-terminal is exemplified. The side chain of the lysine on the linker was functionalized with a bromoacetamide group as the reactive handle. A modified reduction/oxidation (redox) method was used for the conjugation process.^33^ The engineered cysteines on the expressed antibodies were capped with endogenous thiols, such as glutathione or cysteine, to form a heterogeneous mixture, which has the potential to render inconsistent conjugation reaction results. In our investigation of methods to generate a homogeneous construct, we found cysteamine as an effective agent to exchange the endogenous thiols and cap the engineered cysteines.^33^ The cysteamine-capped cys-mAb constructs significantly improved conjugation reaction yields and the purity of the product. In Figure 1B the cysteamine-capped GIPR E384C Ab **5** was treated with an excess of reducing reagent triphenylphosphine-3,3′,3″-trisulfonic acid trisodium salt (TPPTS) to reduce the engineered cysteamine disulfides. After removal of excess TPPTS via desalting, a mild oxidant, dehydroascorbic acid (DHAA), was added to reform the interchain disulfides, and only the engineered cysteines were available for alkylation with the GLP-1 peptide analogue. The resulting solution of antibody **6** was treated with the alkylation reagent **4** to afford the GIPR-Ab/GLP-1 conjugate. Using this method, we were able to conjugate D88C, E384C, and T487C GIPR antibodies efficiently up to gram scales and obtain bivalently conjugated antibodies in over 60% isolated yield after purification.

### Pharmacokinetic (PK) Study in CD-1 Mice

To assess the integrity of the peptide attached to the GIPR-Ab/GLP-1 conjugate and investigate the effect of the antibody conjugation sites on the PK profile, we prepared D88C, E384C and T487C mGIPR-Ab conjugates (**7**, **8**, and **9**) with a GLP-1 analogue [Aib^8,22^;G^36^]­GLP-1­(7–37) attached via a (G_4_S)_3_K linker, respectively. The peptide was modified with Aib at positions 8 and 22 and Gly at position 36 to improve the metabolic stability while retaining its potency.^34^ The PK study was performed on **7**, **8**, and **9** along with Dulaglutide, a hu IgG4 Fc-GLP-1 peptide fusion, for comparison (Table 1). The conjugates were administered to male CD-1 mice as a single intravenous (IV) injection of 5 mg/kg, respectively, and Dulaglutide was administered at 1 mg/kg. Blood samples for PK analysis were obtained for 1 week after administration. Plasma concentration–time profiles and PK parameters obtained from noncompartmental analysis are presented in Table 1. Among the three conjugates, E384C conjugate **8** exhibited the most favorable PK profile, with a terminal half-life (*t*_1/2,z_) of 5.3 days and systemic clearance (CL) of 15 mL/day/kg. The T487C conjugate **9** demonstrated longer *t*_1/2,z_, and slower CL (2.3 days and 35.8 mL/day/kg, respectively) than the D88C conjugate **7** at 1.9 days and 92.3 mL/day/kg, respectively. Notably, all three GIPR-GLP-1 conjugates showed a superior PK profile to Dulaglutide (*t*_1/2,z_: 1.2 days; CL: 98.0 mL/day/kg) in terms of clearance rate.

**Table 1:** Mean Plasma Pharmacokinetic Parameters of mGIPR-Ab/GLP-1 Conjugates **7**, **8**, and **9**, and Dulaglutide Following Intravenous Administration to CD-1 Mice^a^

compound	Cys mutant	dose (mg/kg)	*N*	*t*_1/2,z_ (day)	CL (mL/day/kg)
7	D88C	5	3	1.9	92.3
8	E384C	5	2	5.3	15.0
9	T487C	5	3	2.3	35.8
Dulaglutide		1	4	1.2	98.0

a Mean pharmacokinetic parameter estimates (intact assay) following single intravenous administration of test articles to CD-1 mice. *N* = number of animals; *t*_1/2,z_ = terminal half-life (calculated from 2 to 7 days); CL = clearance after intravenous administration.

A comparison of intact constructs (mGIPR-Ab with covalently attached GLP-1 analogue) and total constructs (mGIPR-Ab with or without GLP-1 analogue peptides) is shown in Figure 2. To enable mechanistic interpretation of *in vivo* disposition, we developed pharmacokinetic assays to quantify both the intact and the total species. The concentration–time profiles of intact conjugates differed substantially across constructs (Figure 2A) and displayed varying degrees of separation from their corresponding total profiles (Figure 2B). In contrast, the total profiles (Figure 2B) were broadly similar for all constructs, indicating that the disposition of the sum of forms (intact + metabolites) was largely consistent and that the observed differences in intact exposure were driven mainly by the differential stability of the GLP-1 component. These data established the GLP-1 conjugation sites as a key determinant of intact peptide stability, with the rank order: **8** (E384C) > **9** (T487C) > **7** (D88C).

**Figure 2:**
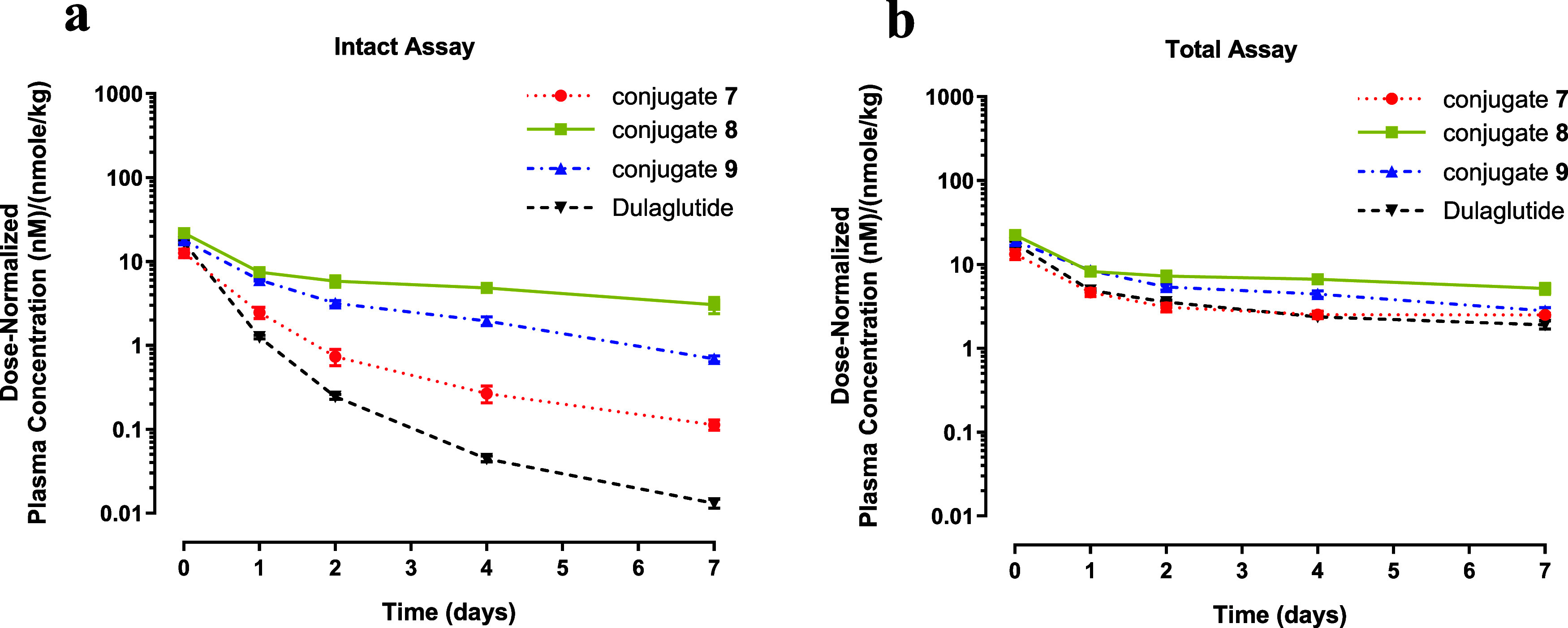
Plasma concentration–time profiles. (a) Dose-normalized mean (±SD) plasma concentration (intact assay) following single IV administration of mGIPR-GLP-1 conjugates (**7**, **8**, and **9**) or Dulaglutide at 5 or 1 mg/kg, respectively, to CD-1 mice in a 1 week study. (b) Profiles of the constructs as in (a), quantified by the total assay. The measurement of intact construct was based on the specificity of the anti-GLP-1 antibody, which binds only when the free N-terminus of the attached GLP-1 component is present. Detection of constructs in the total assay was achieved with a Horseradish peroxidase (HRP)-conjugated mouse monoclonal antibody against human IgG Fc.

### Receptor-Mediated Modulation of cAMP Synthesis

A series of GIPR-Ab/GLP-1 conjugates was prepared by conjugating GLP-1 peptide analogs with various linkers at the three cysteine mutation sites of anti-mGIPR-Ab. The constructs were evaluated for their ability to stimulate cAMP synthesis via GLP-1R and inhibit GIPR activity by displacing GIP in cell-based assays. All tested GIPR-Ab/GLP-1 conjugates were full GLP-1R agonists. Native GLP-1 (compound **1**) activated GLP-1R with an EC_50_ value of 1.5 pM (Table 2). The mGIPR-Ab (compound **2**) and the hGIPR-Ab (compound **3**) inhibited the activity of human GIPR with an IC_50_ of 35.1 and 21.3 nM, respectively. The mGIPR-Ab is significantly more potent in mouse (IC_50_ at 6.2 nM) than the hGIPR-Ab (IC_50_ > 3 uM), so mGIPR-Ab/GLP-1 conjugates were prepared and evaluated in mouse models to optimize the antibody-peptide conjugates.

**Table 2:** GIPR Antagonist and GLP-1R Agonist Activities *In Vitro* ^a^

				**hGLP-1R**	**hGIPR**
	**antibody**	**peptide**	**linker**	**EC**_**50**_ **(pM)**	**IC**_**50**_ **(nM)**
**1**		GLP-1(7–37)-NH_2_		1.5 ± 0.1	
**2**	mGIPR-Ab				35.1 ± 8.0
**3**	hGIPR-Ab				21.3 ± 8.8
**7**	mGIPR-Ab D88C	[Aib^8,22^;G^36^]	(G_4_S)_3_K	6.9 ± 1.6	82.7 ± 16.1
**8**	mGIPR-Ab E384C	[Aib^8,22^;G^36^]	(G_4_S)_3_K	77.3 ± 5.6	26.2 ± 6.9
**9**	mGIPR-Ab T487C	[Aib^8,22^;G^36^]	(G_4_S)_3_K	6.9 ± 1.4	9.7 ± 1.0
**10**	mGIPR-Ab D88C	*c*[E^22^-K^26^;Aib^8^;G^36^]	(G_4_S)_3_K	4.3 ± 1.5	58.1 ± 10.0
**11**	mGIPR-Ab E384C	*c*[E^22^-K^26^;Aib^8^;G^36^]	(G_4_S)_3_K	41.6 ± 3.6	16.6 ± 6.1
**12**	mGIPR-Ab T487C	*c*[E^22^-K^26^;Aib^8^;G^36^]	(G_4_S)_3_K	7.9 ± 1.0	20.2 ± 10.6
**13**	mGIPR-Ab E384C	[Aib^8,22^;G^36^]	K(PEG11)	52.1 ± 17.3	28.8 ± 8.9
**14**	mGIPR-Ab E384C	[Aib^8,22^;G^36^]	AS(AP)_5_GSK	35.5 ± 16.0	23.9 ± 3.1
**15**	mGIPR-Ab E384C	[Aib^8,22^]-GLP-1(7–22)-Ex-4(17–39)	(G_4_S)_3_K	80.0 ± 33.0	14.3 ± 3.9
**16**	mGIPR-Ab E384C	[Aib^8^;E^22^;G^36^]	(G_4_S)_3_K	26.9 ± 9.1	18.7 ± 1.1
**17**	mGIPR-Ab E384C	[Aib^8,22^;E^15^;G^36^]-GLP-1(7–36)	(G_4_S)_3_K	64.2 ± 7.9	23.7 ± 6.3
**18**	mGIPR-Ab E384C	[Aib^8,22^;I^9^;G^36^] GLP-1(7–36)	(G_4_S)_3_K	135.9 ± 6.3	27.2 ± 2.5
**19**	mGIPR-Ab E384C	[Aib^8,22^;A^29^;G^36^] GLP-1(7–36)	(G_4_S)_3_K	564.5 ± 80.0	22.5 ± 4.9
**20**	mGIPR-Ab E384C	[Aib^8,22^;A^31^;G^36^] GLP-1(7–36)	(G_4_S)_3_K	49 ± 7	33.6 ± 11.2
**21**	mGIPR-Ab E384C	[Aib^8,22^;Y^16^;G^36^]	(G_4_S)_3_K	109.1 ± 36.7	12.1 ± 3.3
**22**	mGIPR-Ab E384C	[Aib^8,22^;Y^16^;G^36^]	G_4_SGGK	98.8 ± 9.7	17.0 ± 5.4
**23**	mGIPR-Ab E384C	[Aib^8,22^;K^(linker)26^;G^36^]	(G_4_S)_3_K	36.3 ± 0.6	25.4 ± 2.1
**24**	mGIPR-Ab E384C	[Aib^8^;Y^16^;E^22^;G^36^]	(G_4_S)_3_K	25.0 ± 5.7	17.3 ± 1.3
**25**	hGIPR-Ab E384C	[Aib^8,22^;G^36^]	(G_4_S)_3_K	183.9 ± 45.6	19.3 ± 4.3
**26**	hGIPR-Ab E384C	[Aib^8^;E^22^;G^36^]	(G_4_S)_3_K	45.4 ± 10.7	20.2 ± 4.7
**27**	hGIPR-Ab E384C	[Aib^8,22^;E^15^;G^36^]	(G_4_S)_3_K	280.8 ± 32.9	13.4 ± 3.6
**28**	hGIPR-Ab E384C	[Aib^8^;Y^16^;E^22^;G^36^]	(G_4_S)_3_K	48.7 ± 3.8	21.0 ± 5.3

a EC_50_ values represent conjugate concentrations at which half-maximum activation occurs at the GLP-1R. IC_50_ values represent construct concentrations at half-maximum inhibition that occur at the GIPR. A minimum of three separate experiments were conducted for each construct at the GIPR and GLP-1 receptors. All data are represented as group mean ± SD.

To assess the effect of the conjugation sites on the agonist activity of GLP-1 and antagonist activity of mGIPR-Ab, we conjugated the GLP-1 peptide analogs [Aib^8,22^;G^36^]­GLP-1­(7–37) and *c*[E^22^-K^26^;Aib^8^;G^36^]­GLP-1­(7–37) to the D88C (on VL domain), E384C and T487C (on HC constant domain) mGIPR-Ab mutants, respectively. The antagonistic activity of mGIPR-Ab showed that the D88C conjugates (**7** and **10**) were less potent (82.7 and 58.1 nM, respectively) than the E384C conjugates (**8** and **11**, 26.2 and 16.6 nM, respectively) and T487C conjugates (**9** and **12**, 9.7 and 20.2 nM, respectively). This result indicated that the D88C site was less favorable for linking the peptides, as D88C was close to the complementarity-determining regions (CDRs) and the conjugated peptide could interfere with the CDRs interacting with GIPR. The constructs conjugated at the E384C and T487C sites all exhibited comparable potency (**8**, **9**, **11**, **12**, and **13**–**28**, ranging from 9.7 to 33.6 nM) on inhibition of GIPR, demonstrating that peptides conjugated on the heavy-chain constant domain sites did not impact the interaction of the CDRs with GIPR significantly. *In vitro* GLP-1R agonist activity was also influenced by the antibody conjugation site. Conjugates at D88C (**7** and **10**, 6.9 and 4.3 pM, respectively) and T487C (**9** and **12**, 6.9 and 7.9 pM, respectively) retained sub-10 pM potency, whereas the E384C conjugate (**8** and **11**, 77.3 and 41.6 pM, respectively) showed reduced activity. The loss in potency is consistent with increased steric constraint at the hinge-proximal E384C site. Despite this potency reduction, the E384C site provided the most favorable PK profile relative to T487C and D88C. The *in vivo* pharmacodynamic performance of these site-specific conjugates is evaluated in the following section.

The GLP-1 peptide analog in conjugates **10**–**12** was designed with a cyclic lactam (cyclic E22-K26) in the sequence to stabilize its α-helical structure.^35^ The corresponding constructs (**10**, **11**, and **12**) showed higher GLP-1R agonist potency than the constructs with the linear peptide (**7**, **8**, and **9**), suggesting that stabilizing the α-helical structure could enhance GLP-1R activation. To balance weight loss efficacy with GLP-1R-associated gastrointestinal tolerability, we designed a series of GLP-1 peptide analogs, based on the sequence used in conjugate **8**, that systematically tuned GLP-1R agonist potency by modifying residues involved in receptor engagement. To improve potency, we introduced glutamate, a residue known for its high helix propensity,^36^ at position 22 (**16** and **26** with mGIPR-Ab and hGIPR-Ab respectively) to reduce the helix distortion associated with Gly22 and V16Y mutation (conjugates **21** and **22**, on different linkers) to potentially further stabilize the helical conformation by introducing additional hydrophobic interactions with Phe12 and Leu20 through its aromatic side chain.^37^ We also evaluated an Exendin-4-derived C-terminal segment (Ex-4 (17–39)), which has been reported to improve GLP-1 activity and stability.^38^ These variants all displayed improved or equivalent agonist activity in comparison to that of the parent construct **8**. Notably, the cAMP assay provided limited resolution among the most active molecules, likely due to the overexpression of GLP-1R in the recombinant cell systems compared to endogenous levels. This overexpression can compress apparent potency differences and potentially underestimate more subtle activity shifts. To attenuate the GLP-1R agonist potency, we introduced substitutions expected to weaken receptor engagement, including E9I (**18**), D15E (**17** with mGIPR-Ab and **27** with hGIPR-Ab), I29A (**19**), and W31A (**20**). Among these, mutations I29A (**19**) and E9I (**18**) produced significant reductions in GLP-1R activity, with EC_50_s at 564.5 and 135.9 pM, respectively. In parallel, we evaluated various linkers and found that most of the linkers did not have a significant impact on GLP-1R agonist activity. The rigid linker (**14**)^39^ and PEG linker (**13**) yielded a modest potency improvement, and notably, conjugate **22** with a shorter (GGGGS)­GGK linker maintained the GLP-1R agonist activity.

We also investigated the site of linker attachment to the GLP-1 peptide. Because the N-terminus of GLP-1 is essential for receptor activation, we avoided N-terminal attachment and instead examined the internal conjugation sites. Conjugate **23**, in which a (GGGGS)_3_ linker was attached at Lys26, retained high potency (EC_50_ = 36.3 pM), identifying position 26 as a viable internal attachment site in addition to C-terminal linkage. Finally, to enable efficacy studies in obese NHPs, we conjugated four GLP-1 analogs, [Aib^8,22^;G36], [Aib^8^;E^22^;G^36^], [Aib^8,22^;E^15^;G^36^], and [Aib^8^;Y^16^;E^22^;G^36^] GLP-1(7–37) to hGIPR-Ab E384C to generate constructs **25**–**28**. All four conjugates retained robust activity in both the human GLP-1R agonism and the hGIPR antagonism assays (Table 2).

### *In* *Vivo* Study in db/db Mice

We chose the db/db mouse model of leptin receptor deficiency to evaluate GLP-1R agonist activity of the mGIPR-Ab/GLP-1 conjugates *in vivo* and help us rank the GLP-1 potency and duration based on inhibition of body weight gain and a decrease in blood glucose levels. To compare the antibody conjugation site E384C and T487C, we performed a 12-day multiple dosing study using two sets of conjugates: a linear peptide E384C conjugate **8** versus the corresponding T487C conjugate **9,** and a cyclic E22-K26 peptide E384C conjugate **11** versus the corresponding T487C conjugate **12**. The mice were intraperitoneally (IP) injected with the vehicle, **8**, **9**, **11**, or **12** at 2 mg/kg on study days 0, 4, and 8, and body weight was measured every 1–2 days. The study concluded on day 12 (Figure 3A,B). While vehicle-treated controls gained up to ∼15% body weight, mice dosed with the GIPR-Ab/GLP-1 conjugates maintained a stable body weight with no significant change over the study period. Decreases in blood glucose tracked closely with reduced body weight gain (Figure 3C,D). By the end of the study (day 12), the E384C conjugates showed a greater attenuation of body weight gain than their T487C counterparts (**8** vs **9** and **11** vs **12**). Notably, although the E384C conjugates exhibited lower GLP-1R agonist activities *in vitro* relative to the corresponding T487C conjugates, PK studies indicated that the E384C conjugation produced more stable molecules with a longer half-life. Together, these data suggest that duration of exposure is the dominant driver of *in vivo* efficacy for GIPR-Ab/GLP-1 conjugate; therefore, we selected the E384C conjugation site for evaluation of additional GLP-1 peptide analogs.

**Figure 3:**
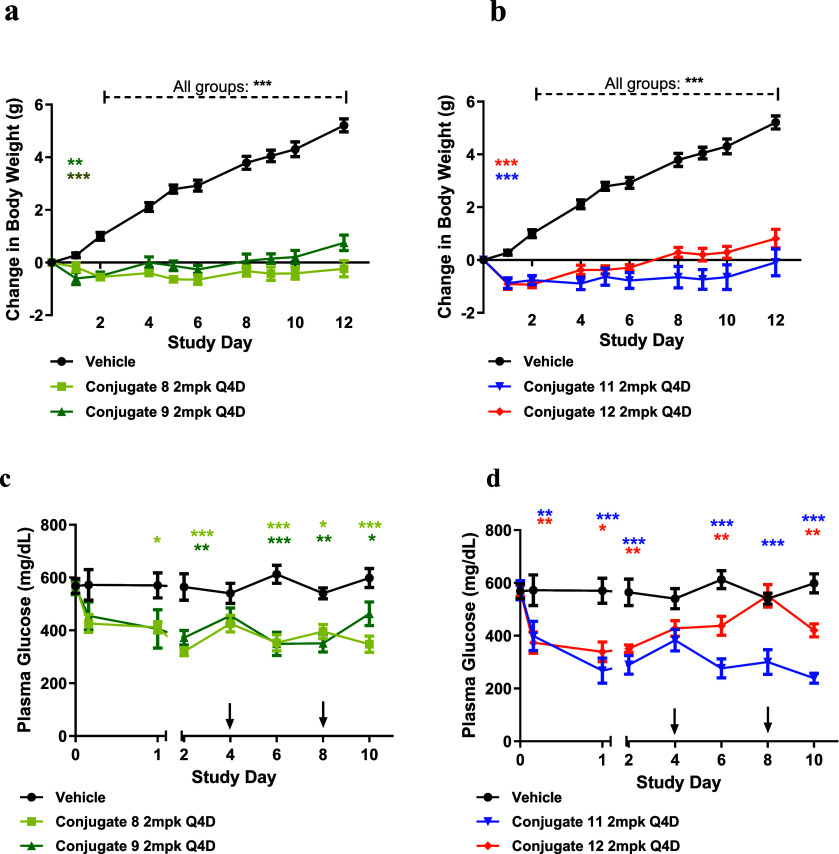
Conjugates **8**, **9**, **11**, and **12** reduced body weight gains in db/db mice. db/db mice were administered vehicle or anti-GIPR/GLP-1 peptide conjugate (2 mg/kg) every 4 days for 8 days. (a, b) Change in body weight was measured over time. (c, d) Plasma glucose level was measured over time. All data are represented as group mean ± SEM *p < 0.05, **p < 0.01, ***p < 0.001 for treatment versus vehicle.

After the antibody conjugation site was selected, we conjugated all the GLP-1 peptide analogs of interest to GIPR-Ab E384C and evaluated *in vivo* GLP-1R agonist activity in db/db mice. Each conjugate was evaluated in a single-dose IP study at 2 mg/kg, with body weight monitored daily throughout the study. In all experiments, conjugate **8** was used as the benchmark. We first evaluated the effect of the peptide conjugation site by comparing the C-term conjugate **8** with a conjugate linked at position 26 (**23**) (Figure 4A). Both constructs attenuated body weight gain relative to the vehicle group; however, the C-term conjugate **8** produced a more pronounced effect than the position 26 conjugate **23**, despite **23** exhibiting higher GLP-1R agonist activity *in vitro*. Based on these findings, we selected the C-term as the preferred attachment site for subsequent GLP-1 peptide analogs. We next examined the effect of linker length using conjugates **22** and **21**, which differ only in the spacing between the antibody conjugation site and the peptide. The longer (GGGGS)_3_ linker conferred more favorable inhibition of weight gain (Figure 4A). Notably, conjugate **21**, incorporating the V16Y substitution to stabilize the GLP-1 α-helical conformation, showed greater efficacy than **8**, which contains the same linker but lacks the V16Y mutation. Among this series, conjugate **24**, with the combination of V16Y and G22E mutations, produced the greatest reduction in body weight (Figure 4A). Consistent with this trend, conjugate **16** with the G22E mutation and GLP-1/Exendin-4 (Ex-4) chimera conjugate **15** also induced greater BW loss than **8** in a separate study (Figure 4B). In contrast, conjugates with D15E (**17**), I29A (**19**), and W31A (**20**) mutations showed a diminished ability to inhibit weight gain. Particularly, the W31A mutation caused a marked loss of GLP-1R agonist activity *in vitro* and resulted in BW gain comparable to the vehicle group (Figure 4C). Conversely, the E9I mutation (**18**) exhibited an effect similar to **8**, indicating that this mutation has a minimal impact on GLP-1R agonist potency *in vivo*.

**Figure 4:**
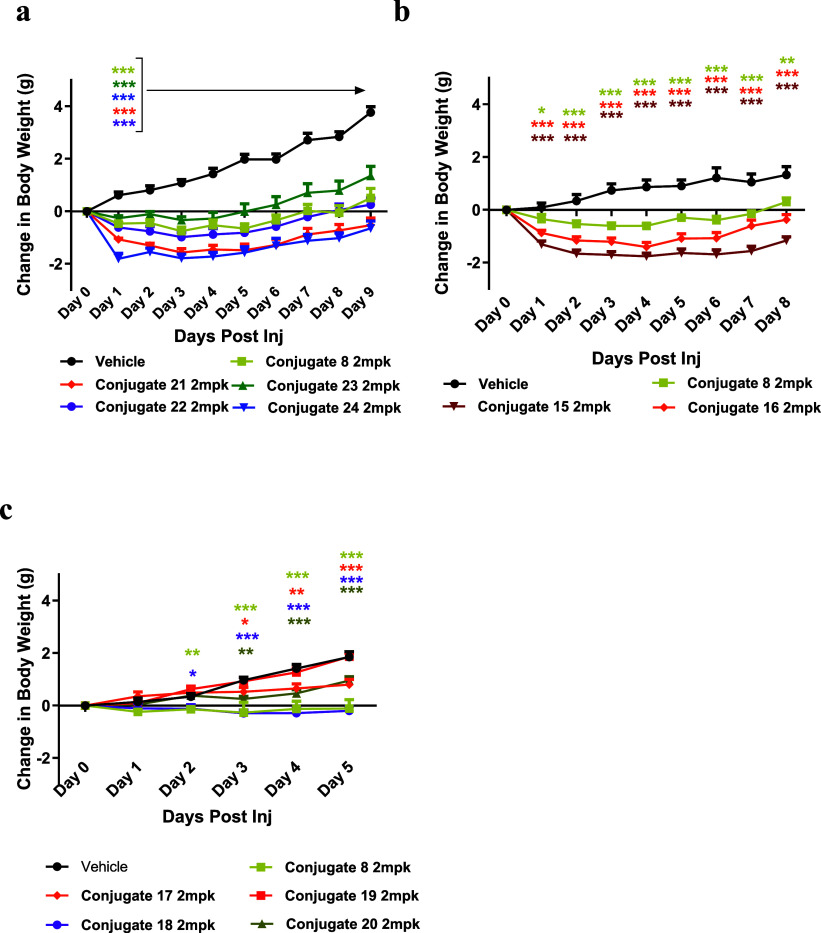
Conjugate **15**–**24** reduced body weight gains in db/db mice (a–c). db/db mice were administered vehicle or GIPR-Ab/GLP-1 peptide conjugate (2 mg/kg). Change in body weight was measured over time. All data are represented as group mean ± SEM *p < 0.05, **p < 0.01, ***p < 0.001 for treatment versus vehicle.

### Efficacy Study in DIO Mice

To further characterize the weight reduction efficacy of mGIPR-Ab/GLP-1 bispecific molecules, we performed a 28-day dose–response study with **8** in DIO mice (Figure 5A). Mice were intraperitoneally (IP) administered with vehicle or different doses of **8** at 0.008, 0.04, 0.2, 1, or 3 mg/kg on day 0 and every 7 days thereafter on days 7, 14, and 21. The study concluded on day 28, 7 days after the previous injection. At low doses of 0.008 and 0.04 mg/kg, conjugate **8** did not show significant weight loss. Starting at the 0.2 mg/kg dose, **8** demonstrated substantial weight loss efficacy, and the effect exhibited a clear dose–response relationship. The group at the dose of 3 mg/kg showed robust weight loss efficacy of 27% on day 28. In the following study, conjugate **8** and **24**, which induced more significant BW loss in db/db mice, were dosed at 0.5 mg/kg in DIO mice on days 0, 6, and 12 (Figure 5B). When the study was concluded on day 18, administration of **8** and **24** led to 16 and 26% BW loss, respectively. Plasma glucose level was significantly reduced to a similar extent in both treatment groups (data not shown).

**Figure 5:**
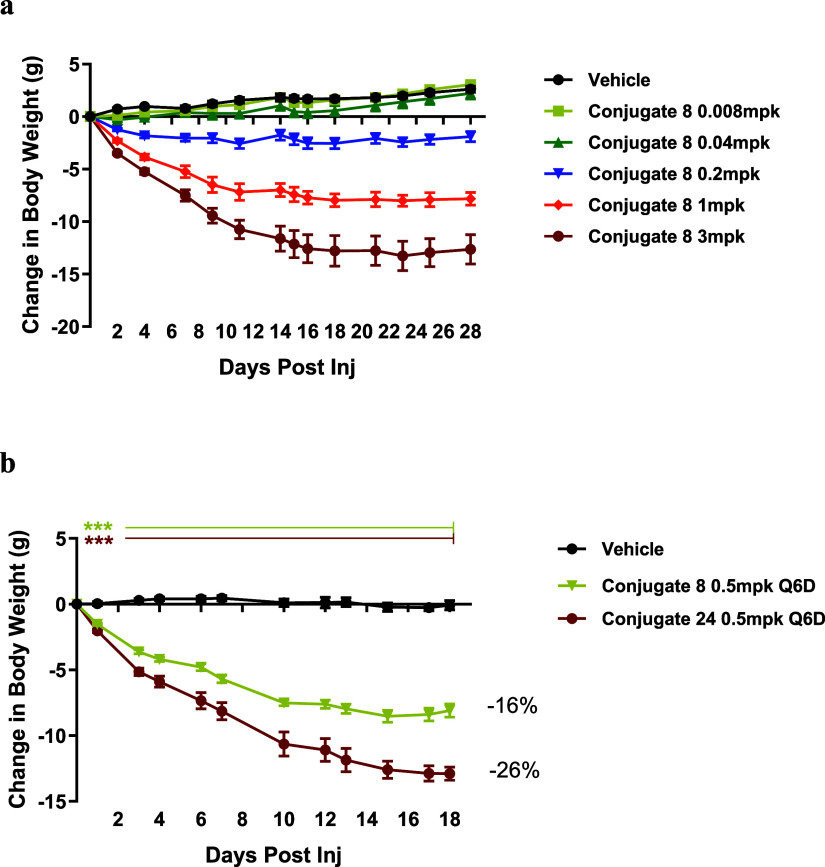
Conjugate **8** and **24** showed BW reduction in DIO mice. (a) Change in body weight was measured over time in DIO mice administered with vehicle, conjugate **8** (0.008, 0.04, 0.2, 1, and 3 mg/kg) every 7 days for 28 days. (b) Change in body weight was measured over time in DIO mice dosed with vehicle, conjugate **8** (0.5 mg/kg), and conjugate 24 (0.5 mg/kg) every 6 days for 18 days. All data are represented as group mean ± SEM *p < 0.05, **p < 0.01, ***p < 0.001 for treatment versus vehicle.

### hGIPR-Ab/GLP-1 Conjugates Reduce Body Weight in Obese Nonhuman Primates

To assess whether the efficacy observed in mice translated to NHPs and to evaluate the impact of the GLP-1R agonist effect on food intake, we first conducted a single-dose tolerability study in obese cynomolgus monkeys. Four GLP-1 analogs, [Aib^8,22^;G^36^] (**25**), [Aib^8,^;E^22^;G^36^] (**26**), [Aib^8,22^;E^15^;G^36^] (**27**), and [Aib^8^;Y^16^;E^22^;G^36^] (**28**) GLP-1(7–37) with the (G_4_S)_3_K linker, were selected for conjugation to hGIPR-Ab. In the db/db mouse study, the relative *in vivo* GLP-1R agonist potency of the corresponding peptide conjugates ranked as Aib^8^Y^16^E^22^G^36^ > Aib^8^E^22^G^36^ > Aib^8,22^G^36^ > Aib^8,22^E^15^G^36^ (Figure 4A,B), enabling evaluation of whether potency-dependent trends observed in rodents would be preserved in NHPs.

Each conjugate (**25**–**28**) was administered as a single subcutaneous dose at 1 mg/kg. Plasma concentrations (Figure 6A) were measured over 28 days, body weight (Figure 6B) was recorded weekly, and food intake (Figure 6C) was monitored daily. All four conjugates showed similar exposure and overall pharmacokinetic profiles (Figure 6A and Table 3), with mean terminal half-life of 9.1, 9.3, 8.8, and 7.9 days for **25**, **26**, **27**, and **28**, respectively, and mean systemic clearance of 11.3, 11.5, 11.2, and 16.1 mL/day/kg, respectively. These PK data indicate that the conjugates were stable in NHPs over the study period.

**Figure 6:**
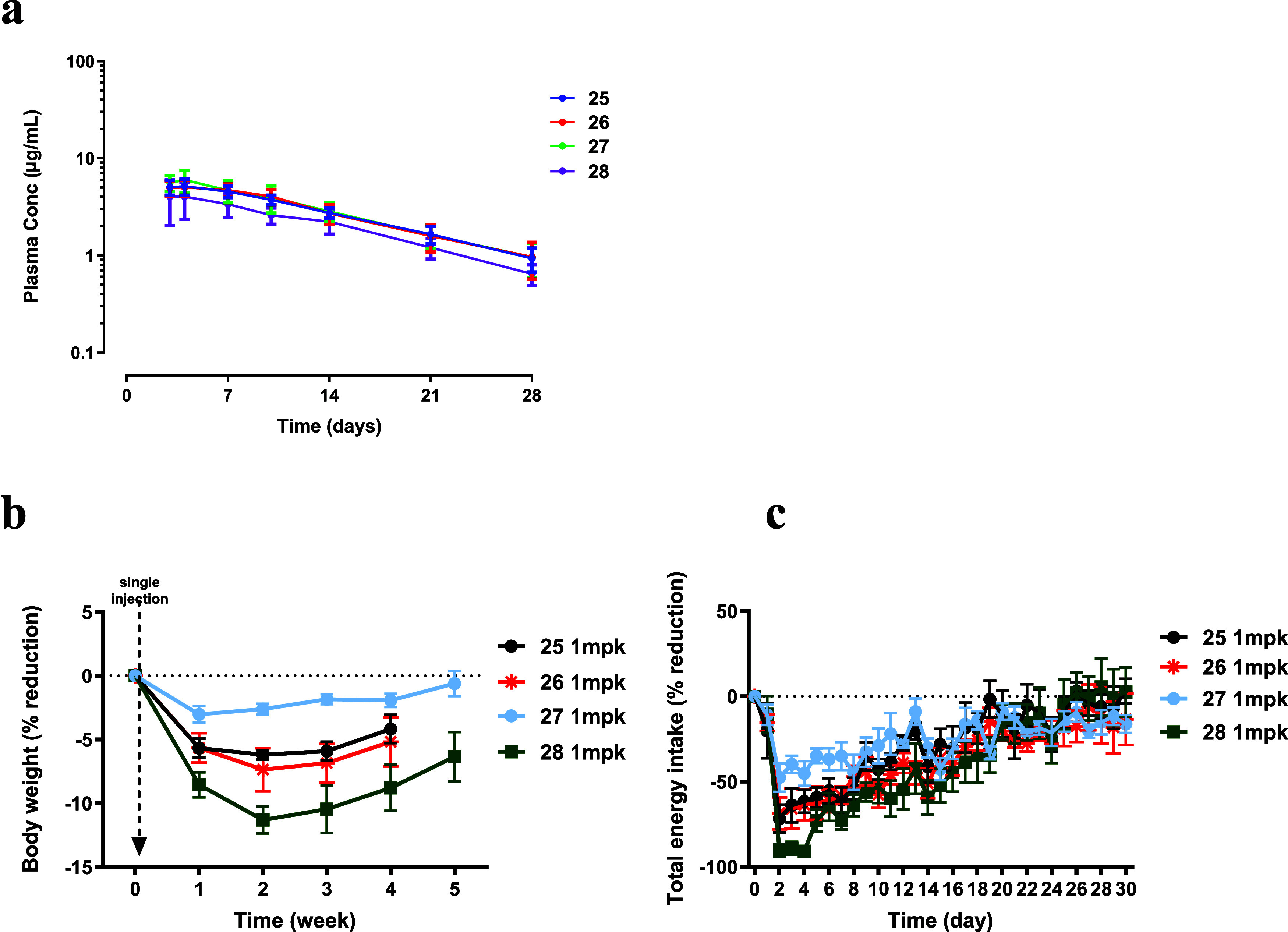
Conjugate **25**, **26**, **27**,and **28** in obese cynomolgus monkey tolerability study. (a) Each group received single subcutaneous injection of **25** (1 mg/kg), **26** (1 mg/kg), **27** (1 mg/kg), and **28** (1 mg/kg), *n* = 5. Exposure measurements started at 3 days post dose and lasted for 4 weeks. (b) Body weight percentage change was measured every week up to 5 weeks. (c) Total energy intake percentage change was measured daily to 30 days. All data are represented as group means ± SEM.

**Table 3:** Mean Plasma Pharmacokinetic Parameters of GIPR-Ab/GLP-1 Conjugates **25**, **26**, **27**, and **28** Following 1 mg/kg Subcutaneous Administration to Obese Cynomolgus Monkeys^a^

compound	*N*	t_1/2,z_ (day)	*t*_max_ (day)	*C*_max_ (μg/mL)	AUC_0‑inf_ (μg day/mL)	CL/F (mL/day/kg)
**25**	5	9.1	4	5.23	89.9	11.3
**26**	5	9.3	4	5.34	91.2	11.5
**27**	5	8.8	4	6.07	94.1	11.2
**28**	5	7.9	4	4.34	65.8	16.1

a Mean pharmacokinetic parameter estimates (intact assay) following single subcutaneous administration of test articles to cynomolgus monkeys. *N* = number of animals; *t*_1/2,z_ = terminal half-life (calculated from 14 to 28 days); *t*_max_ = time of maximum observed concentration (reported as median); *C*_max_ = maximum observed concentration; AUC_0‑inf_ = area under the plasma concentration–time curve from time 0 to infinity; CL/*F* = apparent clearance after subcutaneous administration.

Consistent with the rodent observations, the four conjugates produced differential effects on body weight (Figure 6B). **25**, **26**, and **28** reached maximum weight loss in week two, reducing body weight by approximately 6, 7, and 11%, respectively, whereas **27** reached maximum effect in week 1 with an approximately 3% reduction in body weight (Figure 6B). All four conjugates reduced food intake (Figure 6C), and baseline body weight was not fully regained by the end of the 4 week study period following a single dose. Although the total food and water intake decreased after dosing (data not shown), all four conjugates were well tolerated overall in this study.

Subsequently, the four conjugates (**25**–**28**) were evaluated in a chronic study in obese cynomolgus monkeys by weekly administration at 0.75 mg/kg for 6 weeks (Figure 7). At the end of the treatment phase on day 43, conjugates **25**, **26**, **27**, and **28** decreased body weight by 14.4, 14.5, 8.5, and 16.9%, respectively, compared to the vehicle group. Reduction of food intake was observed after the first dose, and food intake gradually increased during the treatment (Figure 7). Reduction of plasma insulin and lipid levels was also observed.

**Figure 7:**
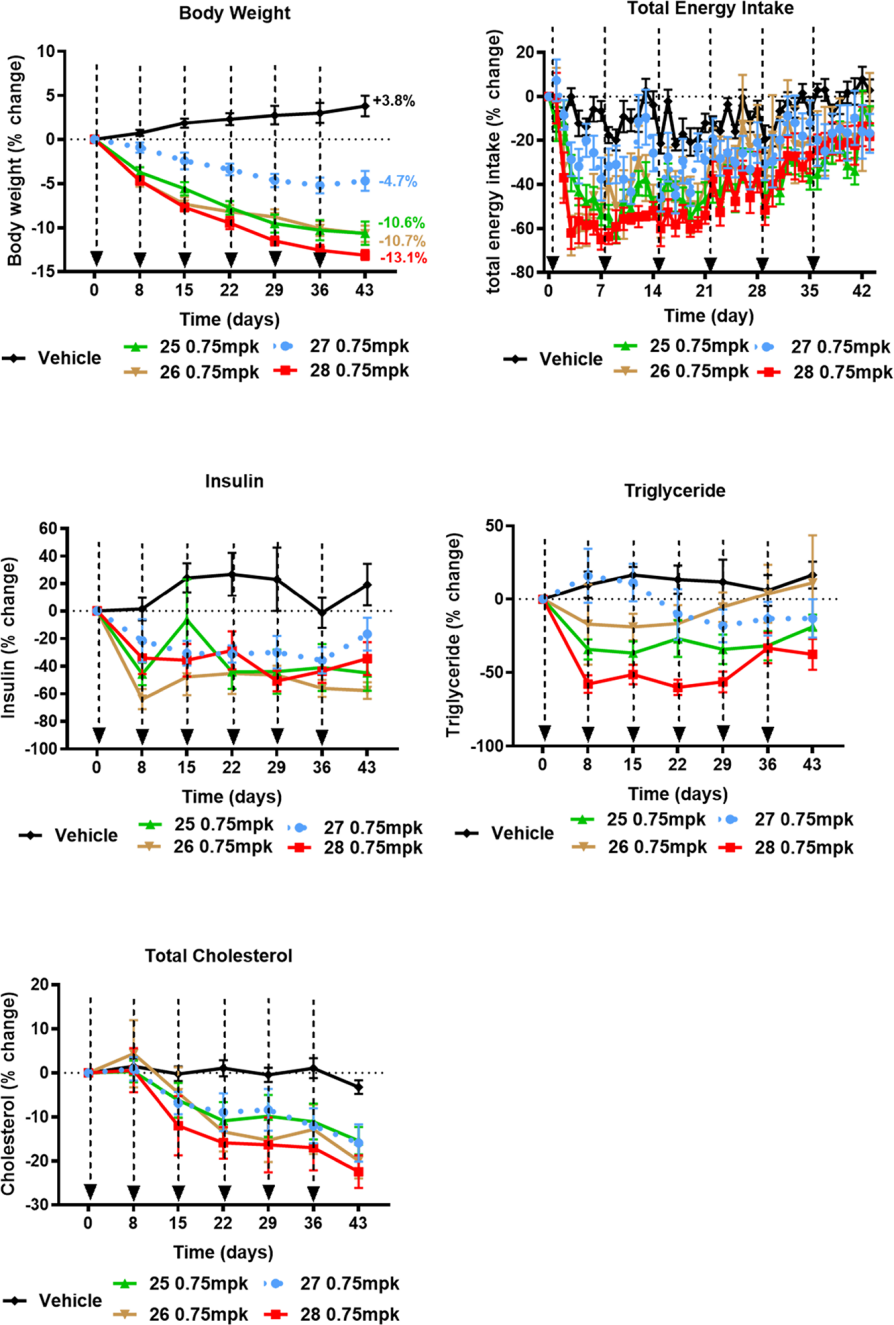
Chronic administration of anti-hGIPR-Ab/GLP-1 peptide conjugates reduced BW in obese monkeys. Effects of anti-GIPR/GLP-1 peptide conjugates on body weight, food intake, insulin, triglycerides, and total cholesterol in obese cynomolgus monkeys after weekly injection (0.75 mg/kg). All data are represented as group mean ± SEM.

## Conclusions and Discussion

Incretin hormones such as GIP and GLP-1 play important roles in glucose homeostasis and have been the focus of type 2 diabetes and obesity treatment development. Lipidated GLP-1 peptide analogs such as Semaglutide and Liraglutide have been approved for chronic weight management. Our earlier report showed that bispecific molecules that bind to GIPR and GLP-1R simultaneously decreased body weight in obese mice and monkeys and improved multiple metabolic parameters.^21^ Further mechanistic investigations demonstrated that the mGIPR-Ab/GLP-1 conjugate targets both central nervous system GIPR and GLP-1R, resulting in additive effects on body weight reduction in obese mice.^40^ Encouraged by the promising results, we set out to further develop a single molecule that targets both GIPR antagonism and GLP-1R agonism pathways for potential clinical development in the treatment of obesity. Our unimolecular antibody-peptide conjugate comprises an anti-GIPR-Ab bearing two copies of GLP-1 peptide analogue attached to engineered cysteine residues via a peptidic linker. A key advantage of this format is that it can confer antibody-like pharmacokinetics, reducing renal filtration and enabling FcRn-mediated recycling, which may support less frequent dosing relative to half-life extension strategies such as lipidation or genetic fusion. In cynomolgus monkeys, conjugation of the peptide to the antibody increased the apparent half-life of the GLP-1 moiety from minutes to over 9 days. As we and others have previously reported, the site of peptide attachment has a significant impact on the stability, PK properties, and functional activity of the conjugates. In the current study, we evaluated three conjugation sites on the anti-GIPR antibody scaffold (two on the heavy chain and one on the light chain). Conjugates with two different GLP-1 peptide analogs attached at D88C or T487C demonstrated approximately 10-fold higher potency than the corresponding E384C conjugates. However, in a subsequent mouse PK study, the E384C conjugate showed the most favorable PK profile with a half-life of 5.3 days, whereas the D88C conjugates provided only modest improvement over dulaglutide, a Fc fusion molecule (*t*_1/2_: 1.9 vs 1.2 days). When E384C and T487C conjugates were evaluated in db/db mice, the E384C conjugate produced a greater and more durable body weight loss. Together, these findings highlight that duration of exposure is a dominant driver of *in vivo* efficacy in this series and further underscore the conjugation site as a primary factor on the pharmacokinetic and pharmacodynamic profiles of antibody-drug conjugates.

GLP-1 peptide analogs conjugated to GIPR antibody were designed to improve metabolic stability while enabling tunable GLP-1R agonist activity. The initial GLP-1 analogue used for the conjugation site study (the peptide in conjugate **8**) incorporated two noncanonical amino acids (Aib) at positions 8 and 22 to enhance stability, along with a Gly36 substitution to avoid potential T-cell epitope interaction. Building on this scaffold, we engineered a series of GLP-1 analogs spanning a range of agonist potencies to identify an optimal balance between weight loss efficacy and tolerability. To attenuate GLP-1 activity, we modified the residues that could potentially impact receptor binding. Replacing the negatively charged Glu at position 9 with a hydrophobic residue Ile, produced body weight reduction comparable to **8** in db/db mice, suggesting a minimal impact on *in vivo* potency. In contrast, D15E and W31A modifications resulted in reduced body weight loss efficacy compared to **8**. The most pronounced attenuation was observed in I29A, which caused a 7-fold loss in cAMP production and yielded no meaningful body weight change in db/db mice relative to vehicle controls, indicating this position is sensitive for maintaining functional agonism.

In parallel, we developed a range of GLP-1 peptide analogs aimed at enhancing stability while preserving or improving potency by stabilizing the GLP-1 α-helical conformation that is adopted upon binding the extracellular domain of GLP-1R, as is typical for peptide ligands of class B GPCRs. We were encouraged by conformationally constrained E22-K26 lactam analogs, which improved the mouse pharmacokinetic profile (data not shown) and increased efficacy in db/db mice. However, lactam-containing peptides may introduce additional development complexity. As a development-friendly alternative, we stabilized helicity through substitutions at positions 22 and 16. Replacing Gly22 with Glu, the corresponding residue in Exendin-4 and one associated with higher helix propensity, enhanced *in vitro* and *in vivo* activity. Similarly, substituting Val16 with a tyrosine residue, which is commonly found in the N-capping motifs of other class B GPCR peptide ligands like glucagon and GRF,^41^ preserved GLP-1 agonist activity in cAMP production. Combining Glu22 and Tyr 16 yielded conjugate **28**, which exhibited robust weight loss efficacy in obese monkeys. Additionally, we explored a GLP-1 N-terminus/Exendin-4 C-terminus chimera, which showed favorable weight loss efficacy in mice. However, conjugate **15** displayed considerable PK variability in monkeys (data not shown), and there were reported concerns regarding the immunogenicity risk associated with Exendin-4 analogs.^42^ Accordingly, we did not advance these molecules further.

The db/db mouse model was used during the initial screening to efficiently explore modifications of GLP-1 peptide analogs. DIO mice then served as the standard rodent chronic model to quantify the body weight loss produced by GIPR-Ab/GLP-1 conjugates. In DIO mice, conjugate **8** demonstrated dose-dependent body weight reduction with once weekly dosing. Under the same dosing schedule (0.5 mg/kg), conjugate **24** showed robust weight loss (∼26% vs vehicle by day 18). Based on the range of efficacy observed in rodents, four GLP-1 analogs were selected to conjugate with hGIPR-Ab, and the resulting conjugates (**25**–**28**) were evaluated in obese monkeys. Across the molecules tested, weight loss efficacy in obese monkeys tracked with the trends observed in rodents. In particular, conjugate **28** achieved close to 17% body weight loss in comparison with the vehicle group after 6 weeks of treatment. In addition to weight loss, the hGIPR-Ab/GLP-1 conjugates improved multiple metabolic parameters, including reductions in insulin, total cholesterol, and triglyceride. Based on its robust *in vivo* efficacy, consistent metabolic improvements, and favorable physicochemical attributes, conjugate **28** was selected as the clinical candidate (AMG 133).

In conclusion, we developed an anti-GIPR-Ab/GLP-1 multispecific molecule in a novel antibody-peptide conjugate format that combines GIPR antagonism and GLP-1R agonism for the treatment of obesity. Efforts to further assess the clinical properties of AMG 133 are currently underway.^43,44^

## Experimental Section

### General Procedures

GLP-1 peptide analogues with linkers were prepared by standard fluorenylmethoxycarbonyl (Fmoc)-based solid peptide synthesis using Rink Amide MBHA resin (Peptide International) on an Intavis MultiPep Rsi synthesizer. 20% 4-methylpiperidine in *N*,*N*-dimethylformamide (DMF) was used for Fmoc removal, and 1,3-diisopropylcarbodiimide (DIC)/ Ethyl cyanohydroxyiminoacetate (Oxyma) were used for amino acid coupling. Boc-His­(Trt)–OH was utilized for the final coupling in the sequence. Each residue was coupled with an excess of coupling solution (5.0 equiv), and each coupling reaction was performed twice at each position. The lysine residue was protected with 4,4-dimethyl-2,6-dioxocyclohex-1-ylidene)-3-methylbutyl (ivDde), and the ivDde group was selectively removed with 2% hydrazine in DMF. Bromo acetyl group was introduced with DIC (10 equiv)/bromoacetic acid (20 equiv) in a mixture of methylene chloride and DMF. After synthesis, the crude peptide was cleaved from resin with trifluoroacetic acid: triisopropylsilane:water (90:5:5) and purified with reverse-phase HPLC to give a white solid after lyophilization.

An anti-GIPR-Ab with a specific cysteine mutation at the E384 position (cys-mAb) was incubated with a solution of 2.5 mM cystamine and 2.5 mM cysteamine in 40 mM HEPES buffer, pH 8.2, at 2.5 mg/mL concentration for 15–20 h. The reaction mixture was filtered through a sterile filter and diluted with 100 mM sodium acetate (NaOAc) buffer, pH 5.0. The reaction mixture was purified by cation exchange chromatography, and then buffer exchanged to 10 mM sodium acetate with 9% sucrose, pH 5.2. The resulting solution of GIPR-Ab cys-mAb (6 mg/mL in 10 mM sodium acetate with 9% sucrose) was partially reduced using four equivalents of triphenylphosphine-3,3′,3″-trisulfonic acid trisodium salt (TPPTS) at room temperature (RT) for 60–90 min. The protein was buffer exchanged into clean 10 mM NaOAc and 9% sucrose, pH 5.2. The reduced GIPR-Ab cys-mAb was diluted with 50 mM sodium phosphate buffer containing 2 mM EDTA, pH 7.5, and treated with eight equiv of dehydroascorbic acid (DHAA, BioSynth International) at room temperature for 90 min. Four equivalents of bromoacetyl-GLP-1 analog peptide were added directly to the solution, and the solution was incubated at RT for 15–20 h. The cloudy solution was sterile filtered with 20 mM Tris-HCl, pH 7.0. The reaction mixture was purified by cation exchange chromatography, and then buffer exchanged into 10 mM NaOAc, 9% sucrose, pH 5.2. Purity of all final compounds was 95% or higher, as determined by high-performance liquid chromatography (HPLC). The identities of the antibody-peptide conjugates were confirmed by LC/TOF-MS analysis on Agilent 1260 and 6230 LC/TOF. SEC analysis was performed on an Agilent 1260 bioinert series with a quat pump and degasser with a Tosoh QC-PAK GFC 300 7.8 mm ID x 15 cm column at ambient temperature.

### *In Vitro* cAMP Assay

The culture media for all the cell lines used in this study, including CHOK1 stably expressing human GLP-1R cells or mouse GLP-1R cells, CHO-AMID cells stably expressing monkey or rat GLP-1R cells, HEK 293T hGIPR cells, CHO-AMID mouse or rat GIPR cells, and 293T monkey GIPR cells, have been previously published.^19^ All cells were cultured in humidified incubators maintained at 37 °C and 5% CO_2_.

The GLP-1R agonist activity of GIPR-Ab/GLP-1 peptide conjugates was determined using cAMP production in CHO cells stably expressing human and mouse GLP-1R as previously described.^21^ The cAMP levels were measured in a PerkinElmer Envision plate reader with a fluorescence ratio of 665/620 nm. The GIPR antagonist activity of GIPR-Ab/GLP-1 peptide conjugates was determined using cAMP production in HEK 293T cells stably expressing human or monkey GIPR and CHO-AM1D stably expressing mouse GIPR as previously described.^19^ The fluorescence was measured in a PerkinElmer Envision plate reader, and the cAMP levels were expressed as a ratio of 665/620 nm.

### *In* *vivo* Studies

The db/db mice studies, DIO mice studies, and obese monkey studies were performed as previously described^19,21^ with minor modifications (Supporting Information). Rodent *in vivo* studies were conducted using intraperitoneal (IP) administration to align with an established screening paradigm for antibody–peptide conjugates and to enable consistent comparative ranking across designs under a single route of administration. Dose levels were determined based on benchmark performance and pilot tolerability assessments to ensure adequate exposure for quantifying body weight and metabolic effects, while minimizing excessive acute pharmacological responses.

### Animal Welfare

Mice were housed in groups at an AAALAC, international-accredited facility. Animals were cared for in accordance with the Guide for the Care and Use of Laboratory Animals, Eighth Edition. All research protocols were reviewed and approved by the Amgen Institutional Animal Care and Use Committee (Approved Protocol Number: 2006–00010).

Mice (*Mus musculus*; BKS.Cg-Dock7^m^ +/+ Lepr^db^/J, stock no.000642, male, Jackson Laboratories; or C57BL/6NHsd fed high-fat diet, male, Inotiv) were housed in individual ventilated caging (IVC) or static caging system (Innorack, Innovive or Greenline, Tecniplast) on an irradiated corncob bedding (Envigo Teklad 7097). Lighting in animal holding rooms was maintained on a 12:12 h light: dark cycle, and the ambient temperature and humidity range were at 68 to 79 F and 30 to 70%, respectively. Animals had ad libitum access to pelleted feed (Envigo Teklad Global Rodent Diet-soy protein free extruded, irradiated 2020X or Research Diets Inc. D12492 60% kcal high-fat diet) and reverse-osmosis (RO) chlorinated (2 to 3 ppm) water via an automatic watering system.

Nonhuman primates were housed in an AAALAC, international-accredited facility. Animals were cared for in accordance with the *Guide for the Care and Use of Laboratory Animals*, Eighth Edition. All research protocols were reviewed and approved by the Kunming Biomed International (KBI) Institutional Animal Care and Use Committee and Amgen External Study Ethical Review Committee (Approved Protocol Number: KBI K001116021–01,01).

Obese cynomolgus monkeys (*Macaca fascicularis*, male, BW > 7 kg; BMI > 41 kg/m^2^) were obtained from KBI’s stock colony and were individually housed in stainless steel cages for the duration of the study. Details on the environment, feeding regimen, and water intake were previously described.^19,21^

### Statistical Analysis for Animal Studies

Statistical analyses were performed using GraphPad Prism or Phoenix WinNonlin. Descriptive statistics (mean ± standard error of the mean (SEM)) were used to summarize the results. Statistical significance was defined as *P < 0.05, **P < 0.01, ***P < 0.001, and ****P < 0.0001 using Student’s *t* test and one-way or two-way analysis of variance (ANOVA).

## Supplementary Material



## Notes

Supporting Information and methods including bioanalytical methods and pharmacokinetics and animal studies (PDF)

## Abbreviations

BMI: body mass index
CL: clearance
DHAA: dehydroascorbic acid
DIC: 1,3-diisopropylcarbodiimide
DIO: diet-induced obesity
DMF: *N*,*N*-dimethylformamide
Ex-4: Exendin-4
FcRn: neonatal Fc receptor
GCGR: glucagon receptor
GLP-1: glucagon-like peptide 1
GLP-1R: glucagon-like peptide 1 receptor
GPCR: G protein-coupled receptor
GRF: growth hormone-releasing factor
HFD: high-fat diet
IP: intraperitoneal
ivDde: 1-(4,4-dimethyl-2,6-dioxocyclohex-1-ylidene)-3-methylbutyl
NaOAc: sodium acetate
NHP: nonhuman primate
Oxyma: ethyl cynohydroxyiminoacetate
TPPTS: triphenylphosphine-3,3′,3″-trisulfonic acid trisodium salt
