# Supplementary material for: Discovery of AMG 133, a Glucose-Dependent Insulinotropic Polypeptide Receptor Antagonist and Glucagon-Like Peptide 1 Receptor Agonist Antibody-Drug Conjugate for the Treatment of Obesity
Source: J Med Chem. 2026 Apr 6;69(8):9348–62. doi: 10.1021/acs.jmedchem.6c00032 (PMC13126679; doi:10.1021/acs.jmedchem.6c00032)
Supplement: Supplementary file 1 [file jm6c00032_si_001.pdf]

Supporting Information

Discovery of AMG 133, a Glucose-Dependent Insulinotropic Polypeptide Receptor Antagonist and Glucagon-Like Peptide 1 Receptor Agonist Antibody-Drug Conjugate for the Treatment of Obesity

Bin Wu*[,1], James R. Falsey[1], Chawita Netirojjanakul[1], Brad Herberich[1], Jerry Ryan Holder[1], Kelvin Sham[1], Jennifer Aral[1], Neil Forsythe[1], Kenneth W. Walker[1], Shu-Chen Lu[2], Todd Hager[3], Shanaka Stanislaus[2], Renee Komorowski[2], Larissa Atangan[2], Michal Achmatowicz[4], Dante Romanini[4], Dohan Weeraratne[3], Les P. Miranda[1], Murielle M. Véniant[2], and Yuan Cheng*[,1]

1. Therapeutic Discovery, Amgen Research, One Amgen Center Dr, Thousand Oaks, California 91320, United States
2. Department of Cardiometabolic Disorders, Amgen Research, One Amgen Center Dr, Thousand Oaks, California 91320, United States
3. Translational Safety & Bioanalytical Sciences, Amgen Research, One Amgen Center Dr, Thousand Oaks, California 91320, United States
4. Process Development, Amgen Inc. One Amgen Center Dr, Thousand Oaks, California 91320, United States

To whom correspondence should be addressed: Email: binw@amgen.com or yuanc@amgen.com

Table of Content

Supplementary Materials and Methods……………………………………………………………S2

Supplemental Materials and Methods

Bioanalytical methods and pharmacokinetics

Concentrations of GIPR-Ab/GLP-1 peptide conjugate in mouse and cynomolgus monkey plasma were determined by ELISA methods developed to monitor intact GIPR-Ab/GLP-1 peptide conjugate (GIPR-Ab with attached GLP-1 analog peptides) and total GIPR-Ab/GLP-1 peptide conjugate (GIPR-Ab with or without GLP-1 analog peptides). The analytical range for both assays was from 30 to 2000 ng/ml. The ELISA reagents and assay for quantitation of intact GIPR-Ab/GLP-1 peptide conjugate were consistent with previously published methods.[1,2]

The total GIPR-Ab/GLP-1 peptide conjugate ELISA assay followed a similar procedure as the intact assay except for the detection reagent. Detection of GIPR-Ab/GLP-1 peptide conjugate in the total assay was achieved with an HRP-conjugated mouse monoclonal Ab against human IgG Fc (Clone No 21.1, Amgen Inc., Thousand Oaks, CA). PK parameters were estimated from individual plasma concentration-nominal time data using standard noncompartmental analysis in Phoenix® WinNonlin® version 6.4 (Certara, Princeton, NJ).

Mice

Mice were housed in groups at an AAALAC, international-accredited facility. Animals were cared for in accordance with the Guide for the Care and Use of Laboratory Animals, Eighth Edition. All research protocols were reviewed and approved by the Amgen Institutional Animal Care and Use Committee.

Non-obese mice

Animals used on study were male CD-1 mice 8 to 12 weeks of age and weighing approximately 30 g (Charles River Laboratories, CA). Following a 1-week acclimation, mice received a single intravenous bolus dose of GIPR-Ab/GLP-1 peptide conjugate (5 mg/kg) or dulaglutide (1 mg/kg) via the lateral tail vein. Blood samples were collected by submandibular venipuncture at predetermined time points up to 7 days after the dose. Whole blood was placed into Microvette® 500 µl K3 EDTA plasma separator tubes (20.1341.102, Sarstedt, Newton, NC), gently mixed by 8–10 manual inversions, and centrifuged at 11,500 x *g* at 4°C for 5 min. The resulting plasma was stored at -70 °C (± 10 °C) until analysis.

db/db mice

The *db/db* mouse study was conducted at Amgen Inc. (Thousand Oaks, CA). Male db/db mice on the BKS background were delivered at 8 weeks of age (Jackson Labs, #000642). Upon arrival,

mice were group-housed at four animals per cage and the cages were changed at least twice per week. Animals had *ad libitum* access to irradiated pelleted feed (Envigo Teklad Global Soy Protein-free Extruded Rodent Diet [2020x]). Animals were acclimated to manual hand restraint beginning at 5 days before the study. BW and blood glucose were measured as a pre-screen to exclude mice from the study with low BW or blood glucose levels. On the day of the study, baseline BW and blood glucose were measured. Using the cage mean for blood glucose and BW, the mice were assigned to vehicle or treatment groups by cage (n=4 mice/cage; n=8/group). Vehicle or GIPR-Ab/GLP-1 peptide conjugate (2.0 mg/kg) was subsequently IP injected. BW was measured before injection (0 h) and at 24-h intervals thereafter up to 216 h post-injection. Blood glucose was measured before injection (0 hour), at 4 h and 24 h, and at 24-h intervals thereafter up to 144 h after the injection under *ad libitum* feeding condition. Blood samples were taken from the retro-orbital sinus vein from each conscious mouse and blood glucose was measured using an Alpha-Trak glucometer (AlphaTrak 2, Zoetis, Parsippany, NJ).

DIO mice

The DIO mouse study was conducted at Amgen Inc. (Thousand Oaks, CA). Male C57BL/6 mice were delivered at approximately 4 weeks of age from Inotiv (formerly Harlan Laboratories then Envigo in CA). Animals were group-housed (two to four mice per cage) and following 1 week of acclimation, were fed a high-fat diet (HFD) containing 60% of energy from fat enriched with saturated fatty acids (D12492, Research Diets, Inc., New Brunswick, NJ) for an additional 8 weeks. Mice were then single-housed and continued with the HFD feeding for the remainder of the study. Following a total of 17 weeks of HFD feeding, mice were acclimated to manual hand restraint and baseline blood glucose levels were measured (day -3). Mice were assigned into vehicle and treatment groups using blood glucose (day -3) and BW (day 0) values as stratification criteria (single housed mice; n=8/group). Vehicle, GIPR-Ab/GLP-1 peptide conjugate was IP injected into 22-week-old male DIO mice every 6 days on study days 0, 6, and 12. The last dose was administered on study day 12 and the study was terminated on study day 18.

Obese cynomolgus monkeys

Nonhuman primates were housed in an AAALAC, International accredited facility. Animals were cared for in accordance with the Guide for the Care and Use of Laboratory Animals, 8th Edition. All research protocols were reviewed and approved by the Kunming Biomed International (KBI) Institutional Animal Care and Use Committee and Amgen External Study Ethical Review Committee.

Obese cynomolgus monkeys (*Macaca fascicularis*, male, BW > 7 kg; BMI > 41 kg/m^2) were obtained from KBI's stock colony and were individually housed in stainless steel cages for the

duration of the study. Details on the environment, feeding regimen and water intake were previously described. [2]

At least 48 naïve male obese cynomolgus monkeys with latest BMI > 41 kg/m2 were pre-selected from KBI colony for single injection tolerability study. Based on veterinary assessment, a total of 43 monkeys were selected to undergo training. After several weeks of training for all study related procedures, 40 out of 43 monkeys were selected based on blood chemistry and BW data collected at the end of the training period and were assigned to 8 groups (n=5 monkeys/group) with equal distribution of blood chemistries and BW. All monkeys received single SC injection. BW was measured weekly; food and water intake were measured daily. Blood was collected for drug concentration only.

At least 95 naïve male obese cynomolgus monkeys with latest BMI > 41 kg/m^2 were pre-selected from KBI monkey colony for chronic multiple injection study. A total of 85 monkeys were selected to undergo training based on veterinary assessment. After several weeks of training for all study-related procedures, 70 out of 85 monkeys were selected based on blood chemistry and BW data collected at the end of the training period. The monkeys were assigned to seven groups (n = 10 monkeys/group) with equal distribution of blood chemistries and BW. Once weekly dosing by SC injection occurred for 6 weeks followed by a 3-week washout period. BW, blood chemistries, and metabolic profiling were measured weekly; food and water intake were monitored daily.

Supplementary References:

1. Lu, S.-C.; Chen, M.; Atangan, L.; Killion, E. A.; Komorowski, R.; Cheng, Y.; Netirojjanakul, C.; Falsey, J. R.; Stolina, M.; Dwyer, D.; Hale, C.; Stanislaus, S.; Hager, T.; Thomas, V. A.; Haroold, J. M.; Lloyd, D. J.; Veniant, M. M. GIPR antagonist antibodies conjugated to GLP-1 peptide are bispecific molecules that decrease weight in obese mice and monkeys. *Cell Rep. Med.* **2021**, *2*, No.100263.
2. Killion, E. A. ; Wang, J. ; Yie, J. ; Shi, S. D.-H. ; Bates, D. ; Min, X. ; Komorowski, R. ; Hager, T. ; Deng, L. ; Atangan, L. ; Lu, S.-C. ; Kurzeja, R. J. M. ; Sivits, G. ; Lin, J. ; Chen, Q. ; Wang, Z. ; Thibault, S. A. ; Abbott, C. M. ; Meng, T. ; Clavette, B. ; Murawsky, C. M. ; Foltz, I. N. ; Rottman, J. B. ; Hale, C. ; Veniant, M. M. ; Lloyd, D, J. Anti-obesity effects of GIPR antagonists alone and in combination with GLP-1R agonists in preclinical models. *Sci. Transl. Med.* **2018**, *10*, No. eaat3392
